# Transient binding and jumping dynamics of p53 along DNA revealed by sub-millisecond resolved single-molecule fluorescence tracking

**DOI:** 10.1038/s41598-020-70763-y

**Published:** 2020-08-13

**Authors:** Dwiky Rendra Graha Subekti, Agato Murata, Yuji Itoh, Satoshi Takahashi, Kiyoto Kamagata

**Affiliations:** 1 Institute of Multidisciplinary Research for Advanced Materials, Tohoku University, Katahira 2-1-1, Aoba-ku, Sendai, 980-8577 Japan; 2 Department of Chemistry, Graduate School of Science, Tohoku University, Sendai, 980-8578 Japan

**Keywords:** Single-molecule biophysics, DNA, Fluorescence spectroscopy, Single-molecule biophysics, Transcription factors, Tumour-suppressor proteins

## Abstract

Characterization of the target search dynamics of DNA-binding proteins along DNA has been hampered by the time resolution of a standard single-molecule fluorescence microscopy. Here, we achieved the time resolution of 0.5 ms in the fluorescence microscopy measurements by optimizing the fluorescence excitation based on critical angle illumination and by utilizing the time delay integration mode of the electron-multiplying charge coupled device. We characterized the target search dynamics of the tumor suppressor p53 along nonspecific DNA at physiological salt concentrations. We identified a short-lived encounter intermediate before the formation of the long-lived p53–DNA complex. Both the jumps and the one-dimensional diffusion of p53 along DNA were accelerated at higher salt concentrations, suggesting the rotation-uncoupled movement of p53 along DNA grooves and conformational changes in the p53/DNA complex. This method can be used to clarify the unresolved dynamics of DNA-binding proteins previously hidden by time averaging.

## Introduction

Sequence-specific DNA-binding proteins bind to their respective target DNAs accurately and quickly and participate in the control and maintenance of cellular functions. To facilitate the search for target sequences among enormous lengths of DNA, these proteins utilize a strategy called facilitated diffusion, which involves a combination of various dynamics, such as one-dimensional (1D) diffusion and jumping along nontarget DNA, three dimensional (3D) diffusion between two separated sites of DNA, and intersegmental transfer at the contact points of two DNA strands^1–6^. Inhibiting the search for and binding to the target DNA by these proteins will lead to insufficient control of cell functions, which could result in disease^7,8^.

The prevailing method for the characterization of the target search dynamics of sequence-specific DNA-binding proteins is single-molecule fluorescence microscopy in combination with tethering and stretching of DNA on the surface of flow cells^9–15^. Typically, DNA-binding proteins labeled with a fluorescent dye or a quantum dot are introduced into the flow cell, allowed to interact with the stretched DNA, and excited selectively by highly inclined and laminated optical sheet (HILO) illumination or by total internal reflection fluorescence (TIRF). Fluorescent spots from single proteins are sequentially tracked using imaging detectors, such as electron-multiplying charge coupled devices (EM-CCDs), enabling the characterization of their target search dynamics. The time resolution of the method is determined by two factors. First, detection of fluorescent molecules at a spatial resolution of ~ 40 nm requires the collection of at least 50 photons, which in turn requires a certain period of data accumulation and sets the time resolution. Second, the frame rate of the imaging detectors (typically 10–50/s) also sets the time resolution. The shortest reported time resolution for localization of DNA-binding proteins along stretched DNA is 8 ms at a spatial resolution of 22–42 nm^16^. Thus, the standard setup of fluorescence microscopy can only provide spatial dynamics for single molecules blurred by time averaging over the period determined by the photon number and/or the frame rate.

The time resolution may be improved using higher excitation power to increase the rate of photon emission and limiting the area of the imaging detector to increase the frame rate. The rate of photon detection from single fluorophores can be easily increased by increasing the excitation laser power at the expense of the total observation period owing to photobleaching. The imaging area can be reduced significantly by considering the one dimensionality of the stretched DNA and by restricting the region of interest by a slit. In the standard mode of EM-CCD, fluorescence photons from single molecules are accumulated as electrons in each pixel of the two-dimensional detector, which are sent and read out by an A/D converter one by one. However, by limiting the observation area to one stretched DNA, the time delay integration (TDI) detection mode of the EM-CCD, provided by Hamamatsu Photonics, can enhance the frame rate up to ~ 100 fold and achieve sub-millisecond detection of the dynamics of DNA-binding proteins.

p53 is a transcription factor that suppresses the cancerization of cells and has been investigated as a representative DNA-binding protein demonstrating facilitated diffusion mainly based on single-molecule fluorescence measurements^8,17–24^. p53 binds to DNA nonspecifically and slides along DNA to search the target DNA sequence^17–19^. p53 possesses two sliding modes having different contacts with DNA by two DNA binding domains: core and C-terminal (CT) domains^20,21^. The disordered linker enables switching between the two sliding modes^22^. p53 slides along DNA rotationally following the DNA groove^15,25^. Target recognition by the sliding p53 is quite low and can be regulated by single mutations^8^. In addition to the sliding, p53 can utilize the ultrafast intersegmental transfer between two DNAs, which could help to skip obstacles bound to DNA during the target search in cells^23^. These dynamics of p53 were partly supported by molecular dynamics simulations^26–29^. Thus, single-molecule fluorescence studies have improved our understanding of the facilitated diffusion of p53.

Despite extensive studies on the facilitated diffusion of p53, the time resolution of these previous studies, several tens of milliseconds, hampers the detection and characterization of various events expected to occur in shorter time frames. First, the 1D diffusion of p53 in the presence of physiological salt concentrations could not be investigated at this time resolution, except in a single study^18^. Because DNA-binding proteins, including p53, dissociate from DNA rapidly at higher salt concentrations due to weakened electrostatic interactions, lower salt concentrations, such as 50 mM KCl, are typically used for the observation of p53 diffusion along DNA^20,22^. However, improvement of the time resolution may enable characterization of 1D diffusion under physiological salt conditions. Second, transient binding intermediates, which are expected to form after the collision of the DNA-binding proteins and DNA before the formation of the tight binding conformation, could not be characterized using the previous time resolution. Although such transient binding intermediates have sometimes been detected using two-protein systems interacting with each other, the intermediate in the DNA–protein systems has been reported only in one example, *lac* repressor^30^. Third, direct observation of the jumping of DNA-binding proteins along DNA, reported only for EcoRV^31^ to date, is extremely difficult at the available time resolution. Accordingly, sub-millisecond detection will enable us to identify the unresolved dynamics of many DNA-binding proteins.

In this study, we optimized a single-molecule fluorescence detection method for DNA-binding proteins along DNA so as to achieve the sub-millisecond time resolution. Using this optimized setup, we characterized the dynamics of p53 at the time resolution of 500 μs. We succeeded in clarifying the salt-dependent 1D diffusion of p53 along DNA, the transient binding intermediate, and the jumping along DNA. The current setup will enable single-molecule characterization of the fast dynamics of other DNA-binding proteins.

## Results

### Optimization of a sub-millisecond detection setup for molecules moving along DNA

**Figure 1:**
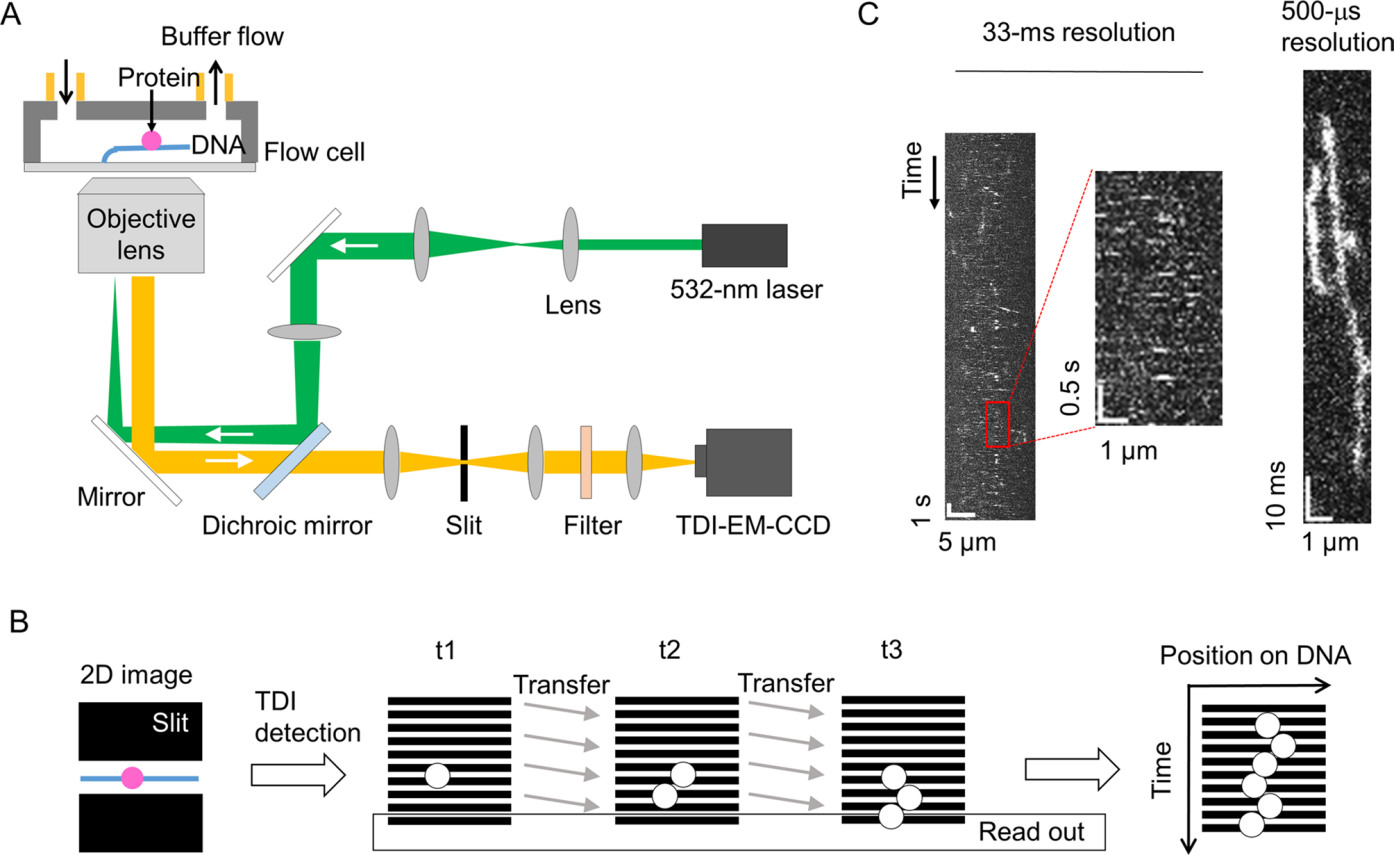
Sub-millisecond-resolved single-molecule fluorescence microscope setup for investigation of DNA-binding proteins interacting with DNA. (**A**) Schematic diagram of the current fluorescence microscopy method. An excitation laser at 532 nm was introduced through an objective lens into a flow cell using HILO, critical-angle TIRF, or TIRF geometry. The fluorescence from the molecules associated with a stretched DNA tethered in the flow cell was collected using the same objective, selected by a slit and optical filters, and detected using an EM-CCD operated at the TDI mode. (**B**) The TDI detection protocol for evaluation of fluorescence from DNA-binding proteins interacting with DNA. The fluorescence photons from a molecule bound to DNA selected by the slit (left) were recorded as charges stored in the rectangle area of the EM-CCD, and the data were transferred line by line and read out (middle). A kymograph was constructed by stacking the transferred 1D images sequentially (right). (**C**) Comparison of kymographs of p53 diffusing along DNA detected by the conventional system operated at a time resolution of 33 ms (left and middle) and by the newly developed system at a time resolution of 500 μs (right). The experiments were conducted in a solution containing 125 mM KCl.

To increase the time resolution of single-molecule fluorescence detection, we combined high-power excitation based on the HILO and/or TIRF geometry of the fluorescence microscope and high-speed line detection based on the TDI mode of the EM-CCD (Fig. 1A). To detect a sufficient number of fluorescence photons in a sub-millisecond time period, we utilized an excitation laser power of 50 mW, corresponding to a power density of ~ 3,000 W/cm^2^ in the epi-geometry with an observation radius of 23 μm. The illumination intensity was 100-fold higher than that of our previous video-rate measurements^8,20^. The DNA was tethered on the optical substrate at one end using the DNA garden method and was stretched by a flow^32^. A rectangular slit in the detection pathway was used to select the fluorescence photons emitted from the labeled proteins bound to a single DNA of interest (Fig. 1B). The photons were detected within the rectangular area (600 × 10 ~ 19 pixels) of the EM-CCD operated in the TDI mode, in which the signal charges were continuously transferred and read out at sub-millisecond intervals (Fig. 1B). The slit prevented the incidence of unexpected photons from the area outside of the single DNA of interest. Therefore, movement of the molecules along the DNA could be directly recorded as a kymograph.

To test the performance of this setup, we measured p53 tetramers labeled with on average 3.2 fluorophores, ATTO532, interacting with the stretched nonspecific DNA^18,20^. Using HILO illumination at a laser power of 0.5 mW and the conventional detection mode of the EM-CCD operated at an interval of 33 ms, we obtained a series of two-dimensional images of the surface of the flow cell, in which more than 10 DNAs were tethered and into which the labeled p53 in the presence of 125 mM KCl was introduced. After the measurement, we chose one DNA area and constructed a kymograph by stacking the one-dimensional images in the order of the measurements. We detected many white dots, not traces, in the kymograph, indicating that many molecules of p53 dissociated from DNA within one frame (33 ms) after binding (Fig. 1C, left and middle panels). In contrast, using the increased laser excitation at 50 mW based on the illumination configuration close to the critical angle (explained below) and TDI image recording, we obtained kymographs at a resolution of 500 μs for p53 under the same solution conditions (Fig. 1C, right panel). In the sub-millisecond resolved kymograph, white traces, not dots, were observed that lasted from several milliseconds to several tens of milliseconds. Additionally, we found that p53 moved along DNA during the duration of its attachment. Accordingly, the optimized setup enabled us to detect the movement of p53 along DNA at sub-millisecond time resolution.

**Figure 2:**
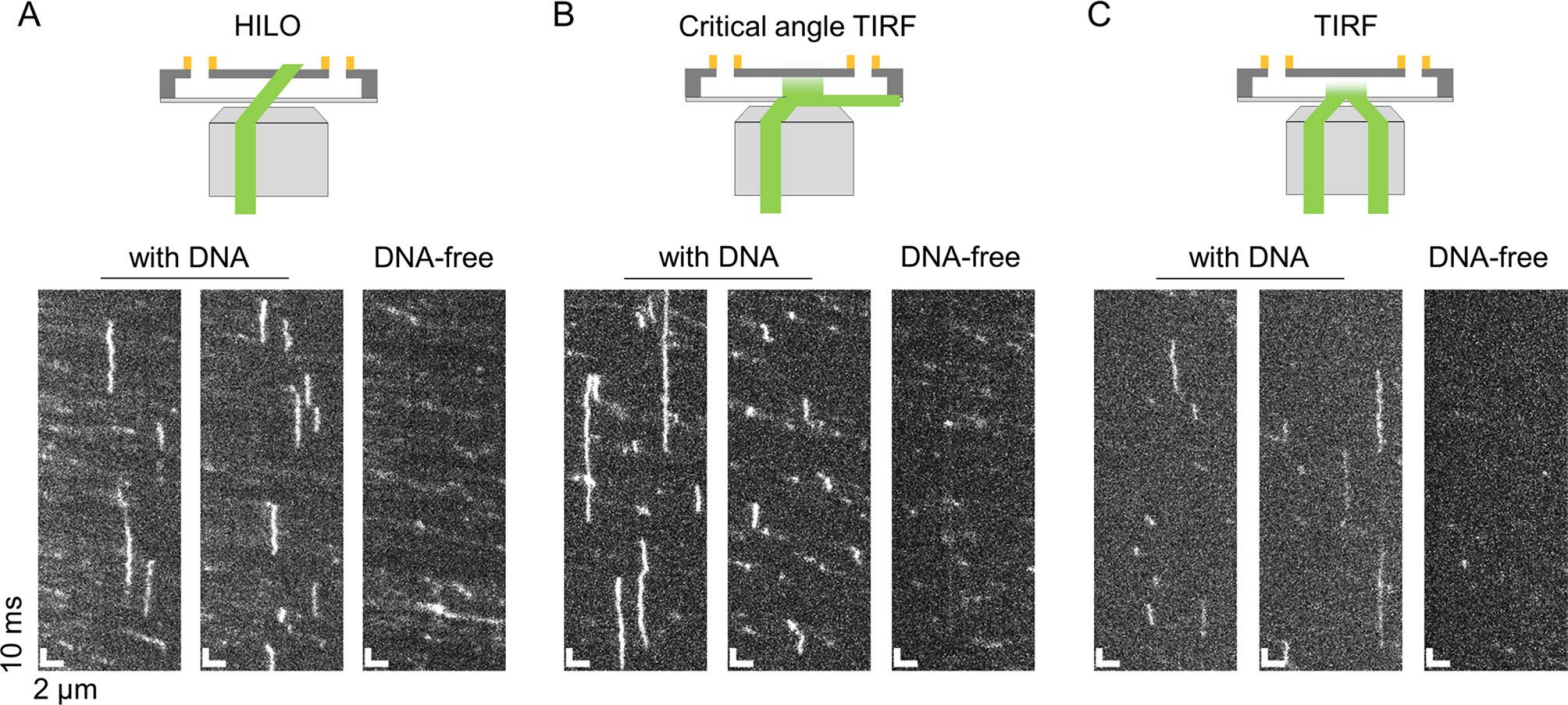
Comparison of three illumination methods suitable for sub-millisecond resolved kymograph measurements of DNA-binding proteins. Typical kymographs of p53 in 150 mM KCl obtained at a time resolution of 500 μs based on the HILO (**A**), critical-angle TIRF (**B**), and TIRF (**C**) methods. The two kymographs on the left of each panel were obtained in the presence of the tethered DNA. The kymographs on the right were obtained without DNA. A schematic illustration of each of the illumination methods is shown in the top panels.

To determine the illumination suitable for ultrafast kymograph measurements of p53, we compared three illumination configurations: HILO, critical angle TIRF, and TIRF (Fig. 2). TIRF was defined as the configuration in which the incident angle of the excitation light was larger than the critical angle (60.7°), the conditions for the total internal reflection were satisfied, and the approach could selectively illuminate molecules bound to the tethered DNA; however, the excitation intensity decreased dramatically as a function of the distance from the surface. The distance between the tethered DNA and the substrate surface was estimated to be ~ 200 nm^33^. For the TIRF condition shown in Fig. 2, we chose 63.5° as the incident angle. In contrast, HILO was the configuration in which the incident angle (59.6°) was smaller than the critical angle, allowing molecules bound to DNA to be illuminated more effectively; however, the bulk molecules flowing in the excitation area would also be excited. Critical-angle TIRF was a variation of TIRF in which the incident angle (61.4°) was adjusted very close to the critical angle. Under these conditions, the penetration depth of the evanescent wave was 268 nm from the substrate surface and was significantly longer than 137 nm, i.e., that in the current TIRF condition at the incident angle of 63.5° (Supplementary text). The illumination intensity of molecules bound to DNA was 2.0-fold larger than that in the TIRF (Supplementary text). We used the same excitation laser intensity of 50 mW for the three measurements.

In the kymograph obtained by the TIRF setup in the solution containing 150 mM KCl, several vertical traces of p53 were observed only in the presence of DNA, confirming the detection of molecules bound to DNA (Fig. 2C). However, the fluorescence intensity of p53 was low owing to the limited excitation intensity by TIRF. In contrast, in the HILO kymograph, several vertical traces were also observed only in the presence of DNA, whose fluorescence intensity was higher than that obtained by TIRF (Fig. 2A). However, many tilted traces were also detected both in the presence and absence of DNA, suggesting the detection of the flowing molecules. The critical-angle TIRF illumination maintained the high fluorescence intensity of molecules bound to DNA, similar to HILO, and significantly reduced the detection of flowing molecules compared with HILO (Fig. 2B). Accordingly, we concluded that critical-angle TIRF illumination was the best approach for sub-millisecond fluorescence detection of molecules interacting with the tethered DNA.

### Two binding components of p53 to DNA

In the sub-millisecond-resolved kymograph of p53 taken at the critical-angle TIRF, short-term traces were more frequently detected in the presence of DNA compared with that in the absence of DNA, suggesting the short-lived binding of p53 to DNA. To confirm the observation quantitatively, we tracked all traces in the kymograph obtained in the presence of DNA at 150 mM KCl and determined their residence times by developing an automated program. As expected, the residence time distribution of the tracked traces showed double exponential decay (Supplementary Fig. S1A). The same tracking performed for the kymograph obtained without DNA gave a distribution whose occurrence was significant (> 25% of that obtained in the presence of DNA) only at the initial time bin from 2 to 3 ms (Supplementary Fig. S1B). The time constants, obtained by fitting the residence time distribution on DNA except for the initial time bin with double exponentials, were 2.8 ± 0.5 ms (94% ± 1%) and 13 ± 3 ms (6% ± 1%), respectively, corresponding to the short-lived and long-lived binding components of p53 to DNA. The residence time distribution obtained after subtraction of the DNA-free data gave identical fitting parameters within the errors (Supplementary Fig. S1C). Furthermore, it is unlikely that the short-lived component was artificially detected due to the blinking of the dye, because the labeled p53 tetramer possessed 3.2 dyes on average. These results suggested that both the short- and long-lived components could be attributed to the binding of p53 to DNA.

**Figure 3:**
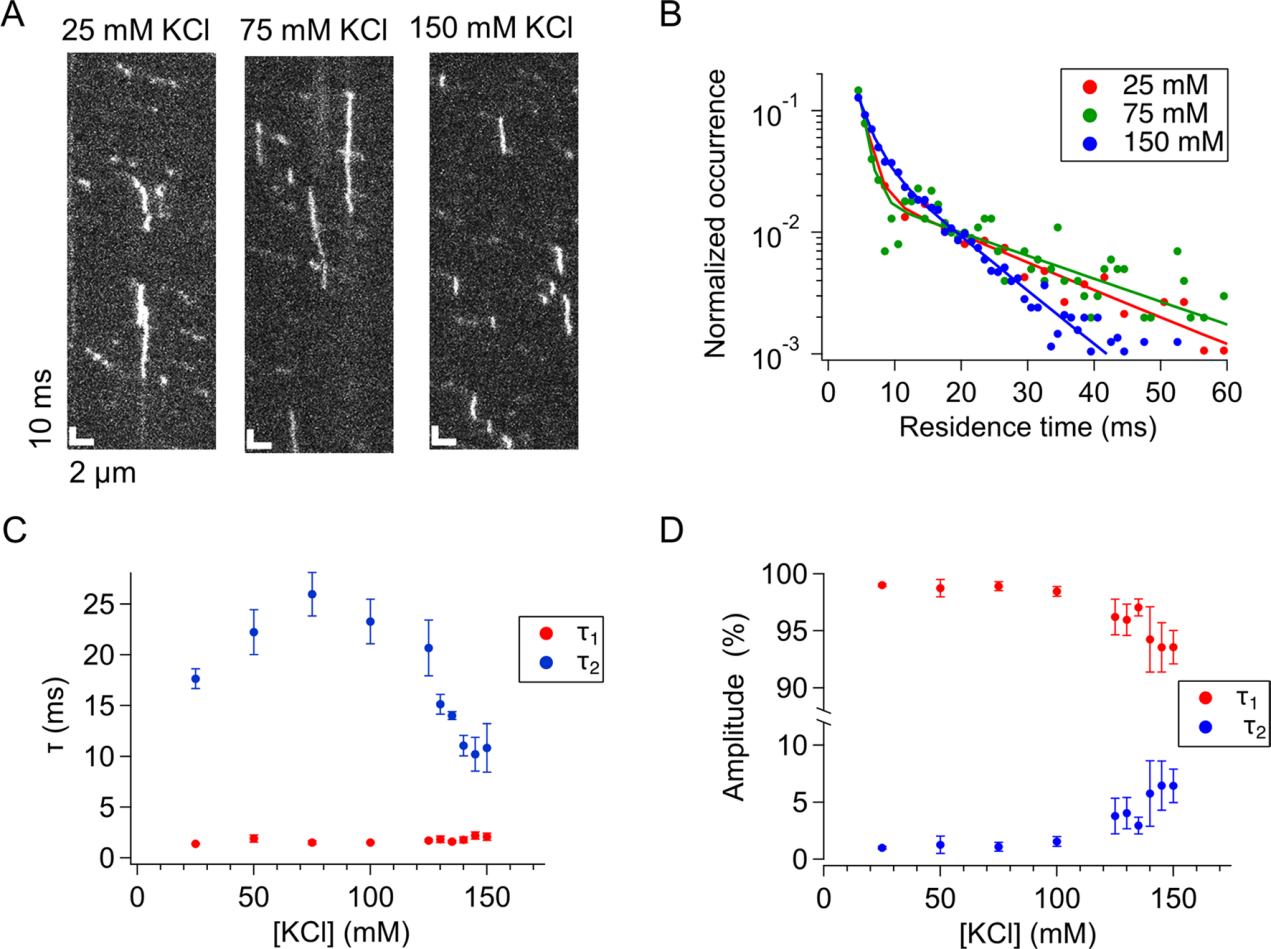
Two components involved in the binding of p53 to DNA. (**A**) Typical kymographs of p53 obtained at the time resolution of 500 μs and at different salt concentrations. (**B**) Residence time distributions of p53 bound to DNA in the presence of different salt concentrations. Solid curves represent the best-fitted curves using the sum of two exponentials. (**C**) Salt-concentration dependence of the time constants obtained by the two exponential fitting. The *τ*_2_ values obtained in the presence of 140, 145, and 150 mM KCl were statistically different from that in 50 mM KCl based on the two-tailed *t* test (*p* < 0.05). (**D**) Salt-concentration dependence of the amplitudes obtained by the two exponential fitting. The errors in panels (**C**,**D**) denote the standard errors calculated from the results of at least three measurements.

To further elucidate the properties of the two binding components, we conducted sub-millisecond-resolved kymograph measurements of p53 at different salt concentrations. As the salt concentration decreased, traces of p53 having extended residence times increased (Fig. 3A), as clearly demonstrated in the residence time distribution (Fig. 3B). The residence time distributions obtained at all salt concentrations could be fitted well by the double exponential functions, whose time constants and amplitudes are presented in Fig. 3C,D, respectively. The time constant of the short-lived component did not depend on the salt concentration (Fig. 3C). The fitted time constant for the long-lived component was constant in the KCl concentration range from 25 to 135 mM, but decreased significantly at KCl concentrations of more than 140 mM. In contrast, the ensemble stopped-flow measurements demonstrated that the residence time of p53 bound to short 30-bp DNA, likely corresponding to the long-lived component, gradually decreased as the KCl concentration increased from 50 to 125 mM (Supplementary Fig. S2). The contradiction of the time constants for the long-lived component in 25–100 mM KCl could be attributed to photobleaching of the fluorescent dye. We confirmed that the photobleaching of the fluorescent dye at higher excitations shortened the apparent residence time of p53 (Supplementary Fig. S3). Also, shorter residence times for 30-bp DNA in 125–150 mM KCl might be caused by the sliding of p53 off from the DNA ends^34^. Furthermore, the fraction of the two components was slightly dependent on the salt concentration when the concentration of KCl was greater than 100 mM (Fig. 3D). The presence of the short-lived component at all salt concentrations suggested that the short-lived component may be an indispensable intermediate to form the long-lived component. Thus, p53 may first bind to DNA, forming the short-lived encounter complex, and may then dissociate from DNA (~ 95%) or convert into the long-lived component (~ 5%).

### Jumps of p53 along DNA

**Figure 4:**
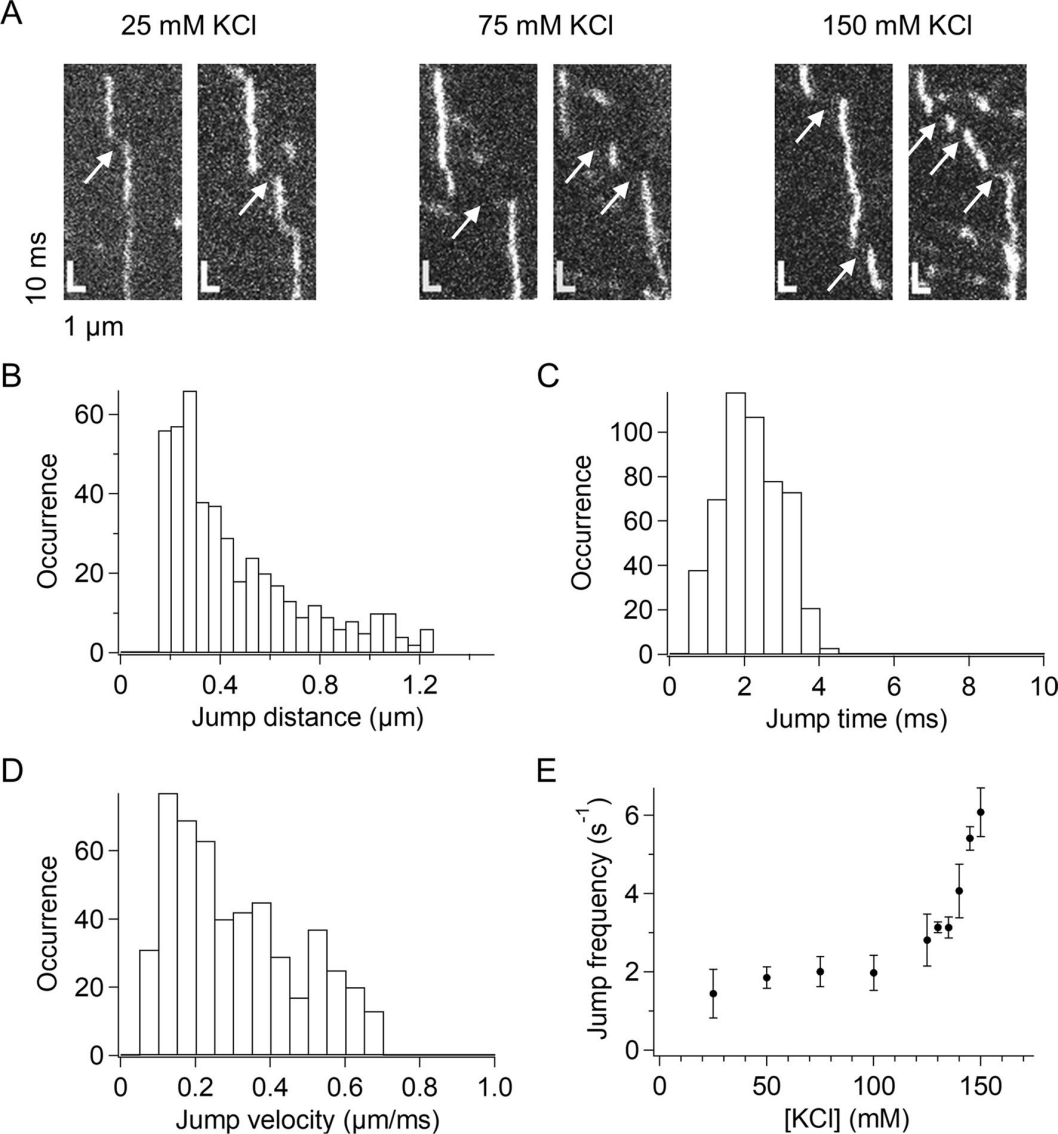
p53 jumped along DNA. (**A**) Typical kymographs of p53 demonstrating traces showing jumps along DNA obtained in the presence of different salt concentrations. Arrows denote the identified jumps. (**B**) Distribution of the jump distance of p53 observed in 150 mM KCl. (**C**) Distribution of the jump time of p53 observed in 150 mM KCl. (**D**) Distribution of the jump velocity of p53 observed in 150 mM KCl. (**E**) Salt-concentration dependence of the average jump frequency of p53. Errors denote the standard errors calculated from at least three measurements. The jump frequencies in 130, 135, 145, and 150 mM KCl were statistically different from that in 50 mM KCl (*p* < 0.05, two-tailed *t* test).

In addition to the short-lived binding component, we noticed another dynamic feature of p53 unresolved in previous studies of video-rate imaging. Specifically, there were sudden shifts in the traces of p53 in the sub-millisecond-resolved kymographs (Fig. 4A). The shifts occurred in the flow direction and were rarely against the flow, suggesting that these shifts represented the transient dissociation of p53 from DNA and its jumping along DNA. The result did not agree with the similar shift frequency in the two directions by assuming that a different molecule binds to DNA immediately after the dissociation of the molecule. The shifts were observed in kymographs obtained at all of the KCl concentrations examined in this study, suggesting that such jump events were a general feature of p53 (Fig. 4A).

To further understand this jumping motion of p53, we selected all jump events from the observed traces based on the following criteria: shifts having a jump distance larger than that expected from the 1D diffusion and shorter than that expected from the bulk flow. The jump distance distribution of the events selected from the kymograph obtained in 150 mM KCl demonstrated a distance-dependent decrease in the re-association probability of p53 (Fig. 4B). Under these conditions, the average jump time and average jump velocity were 2.2 ± 0.2 ms (Fig. 4C) and 0.291 ± 0.007 mm/s (Fig. 4D), respectively. Interestingly, these parameters were not dependent on the salt concentration (Supplementary Fig. S4). By contrast, the jump frequency was dependent on the salt concentration and was enhanced by 3.1-fold in 150 mM KCl compared with that in 100 mM KCl (Fig. 4E). These results implied that the stronger electrostatic interaction between p53 and DNA at lower salt concentrations prevented the transient dissociation of p53 from DNA rather than affecting its re-association with DNA.

### 1D diffusion of p53 along DNA at physiological salt concentrations

**Figure 5:**
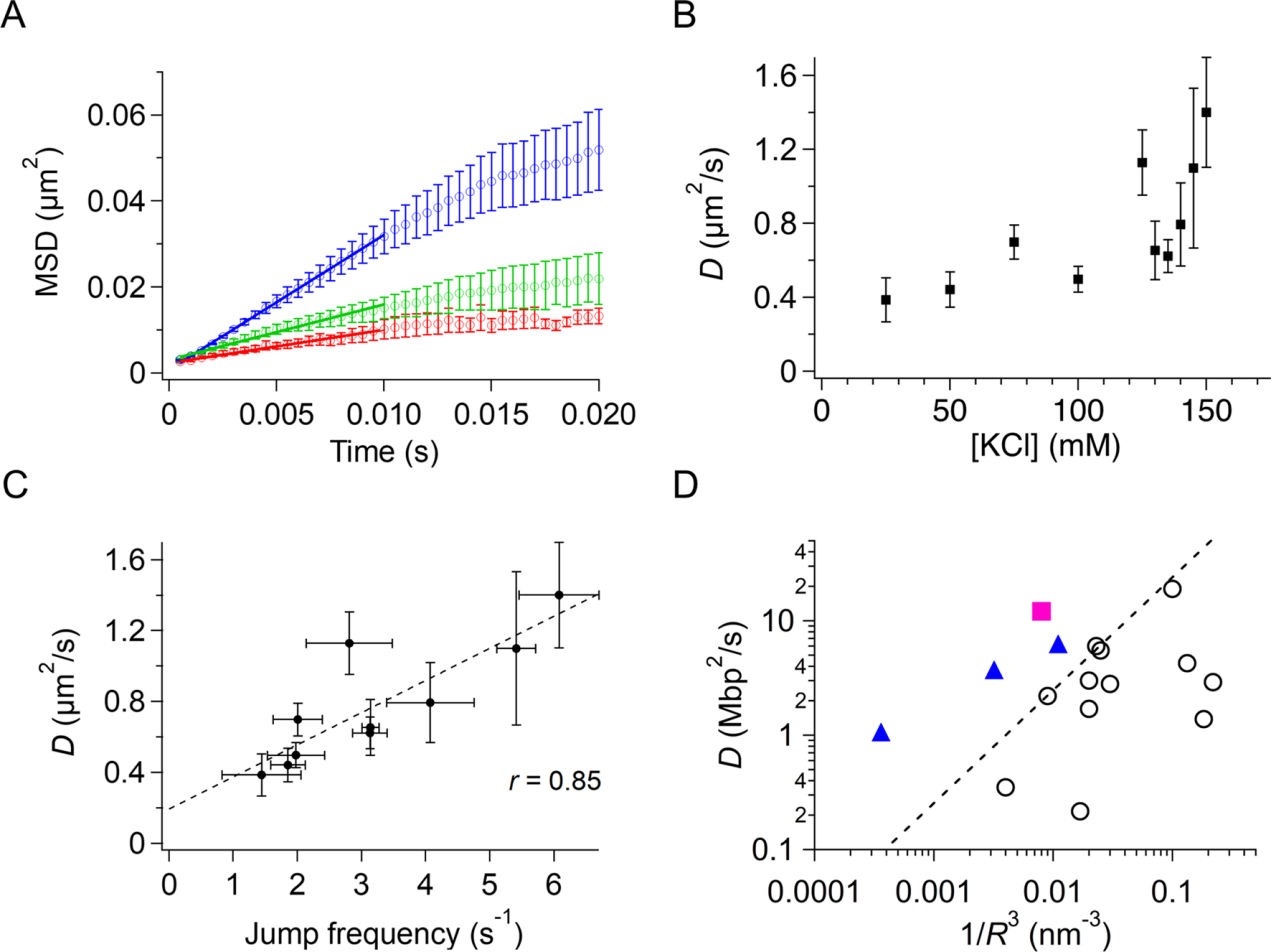
Diffusion of p53 along DNA without following the DNA grooves in the presence of physiological salt concentrations. (**A**) Mean squared displacement (MSD) plots of p53 diffusing along DNA in the presence of different concentrations of salt. Red, green, and blue traces correspond to the plots obtained in the presence of 25, 100, and 150 mM KCl. Straight lines show the best fitted linear functions for the MSD data from 500 μs to 10 ms. (**B**) Salt-concentration dependence of the 1D diffusion coefficient of p53 along DNA. The data obtained in the presence of 125 and 150 mM KCl were statistically different from that obtained in 50 mM KCl (*p* < 0.05, one-tailed *t* test). (**C**) Relationship between the jump frequency and the 1D diffusion coefficient of p53 along DNA in the presence of different concentrations of salt. The dotted line is the best-fitted linear correlation of the two quantities. (**D**) Relationship between the reciprocal of the cube of the radius, 1/*R*^3^, and the 1D diffusion coefficient, *D*, along DNA for various DNA binding proteins. Open circles are data categorized for proteins demonstrating rotation-coupled diffusion along the grooves. The pink closed square denotes the datum for p53 obtained in 150 mM KCl using the current system. Triangles are the data for TALE showing rotation-uncoupled diffusion. The dashed line is the boundary between the rotation-coupled and uncoupled diffusions. In panels (**A**–**C**), the errors denote the standard error calculated from at least three measurements.

The 1D diffusion of p53 along DNA at physiological salt concentrations has never been examined owing to the short residence time of several milliseconds, except in one pioneering study^18^. Accordingly, we next analyzed the 1D diffusion dynamics of p53 detected in the sub-millisecond-resolved kymographs. For all detected traces, we tracked the center of the molecule by fitting the Gaussian function to the fluorescence intensity distribution at each time and obtained the time series of the diffusion dynamics. If a trace contained a jump, we treated the trace as two independent traces separated by the jump, thus eliminating the jump events in the analysis. The average mean square displacement (MSD) of the traces showed a linear increase against time within 10 ms, indicating the diffusional motion of p53 (Fig. 5A). The linearity of MSD was confirmed at all KCl concentrations examined between 25 and 150 mM (Supplementary Fig. S5). The diffusion coefficient, *D*, obtained from the slope of the linear region of MSD, increased gradually as the salt concentration increased (Fig. 5B). The salt-dependent increase in *D* seemed to be coupled with that of the jump frequency (Fig. 4E). In fact, the *D* value was highly correlated with the jump frequency at various salt concentrations (Fig. 5C, *r* = 0.85). These results suggested that hops, not apparent even in the current sub-millisecond measurements, may occur during the 1D diffusion of p53 along DNA. We hypothesized that hops in DNA-binding domains occurred more frequently than the detectable larger jumps during the 1D diffusion, resulting in the enhancement of 1D diffusion at higher salt concentrations.

If the DNA-binding domains of p53 hopped frequently along DNA at higher salt concentrations, 1D diffusion should not occur along the grooves of DNA and should not be coupled with rotation around the DNA. To examine whether the 1D diffusion of p53 at physiological salt concentrations occurred along the DNA groove or not, we plotted the relationship between *D* and the molecular radii of p53 and other proteins, which could differentiate the rotation-coupled diffusion along the DNA groove and the rotation-uncoupled diffusion^14,25,35^ (Fig. 5D). Many proteins were located within the group showing the rotation-coupled diffusion along the DNA groove (open circles). In contrast, the current *D* value for p53 (closed square) obtained in 150 mM KCl was much larger than those of the proteins showing the rotation-coupled diffusion and having the similar size, but was rather in line with those of TALE proteins, showing rotation-uncoupled diffusion (triangles). The results suggested that the 1D diffusion of p53 was not coupled with the major groove in DNA, consistent with our hypothesis that p53 moved along DNA more efficiently at higher salt concentrations by hopping of DNA-binding domains without strictly following the DNA groove.

## Discussion

In this study, we optimized a single-molecule tracking method for DNA-binding proteins along DNA at the time resolution of 500 μs. The sub-millisecond time resolution was achieved using 1D detection based on the TDI mode of the EM-CCD, the slit for the selection of a single DNA of interest, and high-power laser excitation based on critical-angle TIRF. Using the optimized system, we clarified the presence of the short-lived encounter complex of p53 bound to DNA, the jumping of p53 along DNA, and the increased diffusion of p53 at near physiological salt concentrations. These newly characterized dynamics of p53 provide insights into our understanding of the facilitated diffusion of DNA-binding proteins.

Improvement of the time resolution of the current system was achieved through consideration of the fluorescence photon numbers available within a short period of time and using the fast read-out mode of the imaging detectors. The TDI detection was originally developed to capture a moving object by transferring integrated signal charges with the object movement at the same speed so as to check the quality of products in factory. Because the movement of proteins is restricted within stretched DNA, we used the TDI mode of the EM-CCD, provided by Hamamatsu Photonics, and detected 1D images at a time resolution of 500 μs. The TDI mode could potentially reduce the time resolution further to 20 μs. Alternatively, a time resolution of up to ~ 2 ms could be achieved using the standard mode of an EM-CCD by setting a small region of interest.

The other optimized point for the increase in time resolution is the number of available photons in a short period of time. Using the current critical-angle TIRF excitation settings (intensity of 50 mW), we collected 50–200 photons during the time resolution of 500 µs. Currently available microscopes can achieve a spatial resolution of 16–24 nm for a spatially fixed molecule ^36^. This estimate is smaller than the spatial uncertainly of the observed traces (27–72 nm), calculated from the intersection of the MSD plots of p53 at *t* = 0, but is reasonable considering the additional blurring of the current data caused by the fluctuations of DNA. The spatial resolution of the system is comparable to that of previous video rate measurements^16,20^. The increased fluorescent intensity in a short time can also be achieved by the HILO setup but the method detects the bulk molecules flowing in the relatively large excitation area as well as the molecules bound to DNA (Fig. 2). The critical-angle TIRF illumination can reduce the detection number of bulk molecules significantly because of the limited excitation area maintaining the high fluorescence intensity of the bound molecules. In contrast, the TIRF illumination decreases the fluorescence intensity significantly, because the penetration depth of the evanescent wave is smaller than the distance between the bound molecules and the surface. Thus, the critical-angle TIRF setup coupled with the high excitation power enables sub-millisecond fluorescence detection of molecules interacting with the tethered DNA.

**Figure 6:**
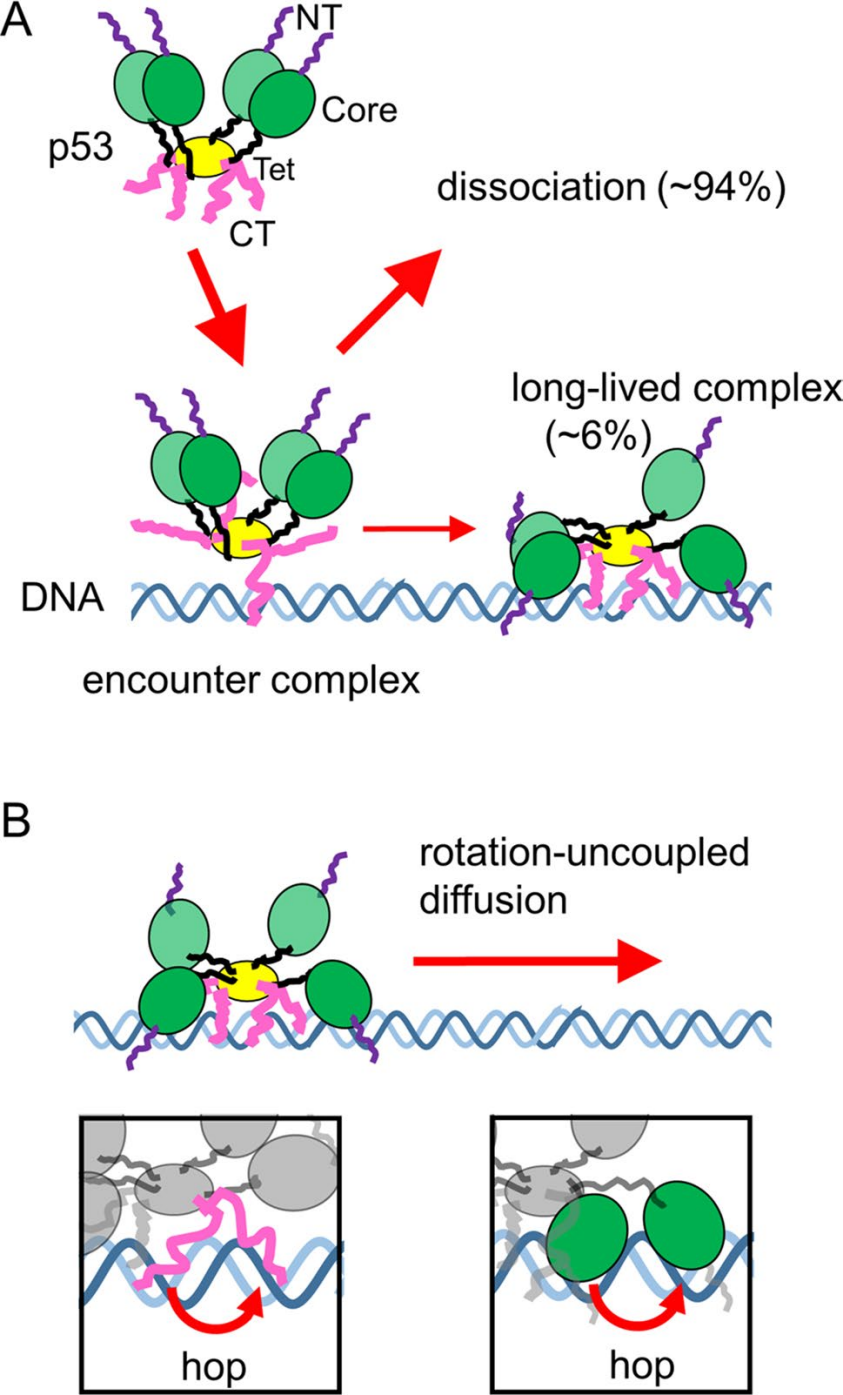
Proposed model of the target search dynamics of p53 based on sub-millisecond-resolved single-molecule measurements. (**A**) Schematic diagram of the encounter and long-lived complexes of p53 tetramer and DNA. NT, Core, Tet, and CT respectively denote the N-terminal, core, tetramerization, and C-terminal domains of p53. At least one of the CT domains interacts with DNA in the encounter complex, and other DNA-binding domains are recruited to form the long-lived complex in the subsequent step. (**B**) Schematic diagram of the rotation-uncoupled motion of p53 in the presence of physiological salt concentrations. The CT and/or core domains hop from one phosphate backbone to the other backbone separated by a half turn of the helix, resulting in rotation-uncoupled diffusion (insets).

In this study, we observed an encounter complex during the association process of p53 to a nonspecific sequence of DNA; this complex had a lifetime of several milliseconds before forming a long-lived complex. Our findings suggested that p53 interacted with DNA loosely in the encounter complex and that changes its conformation increased contacts of DNA-binding domains with the DNA and formed a more stable complex, as required for the target search along DNA (Fig. 6A). Because one p53 tetramer possesses four sets of two DNA-binding domains, the encounter complex could form contacts with DNA via some of the eight DNA-binding domains. Considering the higher affinity of the CT-disordered domain to DNA relative to the core domain, the CT-disordered domain likely participated in the encounter complex^21^. During the next step, the remaining DNA-binding domains may be recruited to form a stable long-lived complex. Interestingly, the conversion rate of the encounter complex to the long-lived complex was 6%, suggesting that the conformational change in p53 occurred rather slowly during the time frame of several milliseconds. Because many DNA-binding proteins possess multiple DNA-binding domains and flexible disordered regions, other DNA-binding proteins may also form encounter complexes similar to that formed by p53.

An encounter complex is an indispensable intermediate required for the formation of a stable complex in the association of two molecules, including protein/ligand, protein/protein, and protein/DNA pairs^37,38^. The encounter complexes were experimentally detected by measuring the concentration dependence of the reaction rate constant because the saturation of the association rate at the higher concentration represents the conversion of the encounter complex to the final product^37^. The *R*_2_ dispersion experiments based on nuclear magnetic resonance (NMR) enabled detection of the encounter complex directly^39^. The observed lifetime of the encounter complex was less than several milliseconds^39,40^. In the p53/DNA system, the encounter complex had a longer lifetime of several milliseconds, as determined by the slow conformational change of p53 from the encounter complex to the long-lived stable complex described above (Fig. 6A).

The current sub-millisecond resolved data demonstrated the significant increase in the 1D diffusion coefficient of p53 along DNA at KCl concentrations higher than 100 mM; however, the data contradicted a previous report showing the independence of the diffusion coefficient against the concentration of monovalent ions^18^. The previous data were obtained using a video-rate system and by analyzing p53 sliding on DNA for extremely long periods, corresponding to rare events with a residence time more than tenfold longer than the ensemble data. The 1D diffusion of such rare molecules could be slower than that of the major populations.

To explain the salt-dependent enhancement of the 1D diffusion, we propose that p53 in the long-lived complex may change its conformation and move along DNA in the rotation-uncoupled manner at high salt concentrations (Fig. 6B). In fact, various physical parameters, including the diffusion coefficient and the jump frequency, changed significantly at 100 mM KCl, supporting the observed conformational changes in the p53/DNA complex. At low salt concentrations, the DNA-binding domains interacted with DNA tightly, making p53 move following the phosphate backbone of DNA. In contrast, because high salt concentrations weaken the interaction between the domains and DNA, the domains may hop from one phosphate backbone of DNA to the other phosphate backbone separated by a half turn of the helix, resulting in the rotation-uncoupled movements of p53. Simultaneous hopping in the domains contacting the DNA may cause the dissociation of p53 from DNA and/or jumping of p53 along DNA.

This model is consistent with the following results by us and other researchers. Molecular dynamics simulations demonstrated that p53 moved along DNA in a rotation-uncoupled manner at high salt concentrations, likely because of weakened interactions between p53 and DNA^27^. The number of DNA-binding domains in contact with DNA decreased as the salt concentration increased^27^. In addition, hopping of the core domain or CT domain on DNA, observed in two independent simulations^26,27^, could enable p53 to transfer between different DNA backbones. This is consistent with the restricted hopping of the core domain in the sliding mechanism proposed based on single-molecule measurements^18^. The correlation between the diffusion coefficient and the jump frequency of p53 implied that simultaneous hopping of domains in contact with DNA may trigger the dissociation of p53 from DNA and may increase the observed jump frequency (Fig. 5C). The 1D diffusion caused by hopping of domains might be affected by the bulk flow (Fig. S6), and further investigation will be required using the stretched DNA in the absence of the flow, for instance, by tethering of two DNA ends to the surface or optical tweezers.

Finally, we discuss the target search mechanism of p53 in cells. The search distance at which p53 moves along DNA per single binding is a key factor determining the search time for the target in cells. The average search distance of p53 was estimated to be 700 ± 100 bp using the diffusion coefficient and residence time in 150 mM KCl. The short residence time (18 ms, corrected by the photobleaching effect as explained in Supplementary text and Supplementary Fig. S1D) was compensated for by the fast 1D diffusion (1.2 × 10^7^ bp^2^/s), demonstrating that the rotation-uncoupled motion promotes the 1D diffusion and contributes to the increased search distance. Interestingly, the estimated search distance was larger than the average distance between two molecules of DNA-binding proteins bound to DNA in cells (less than 100 bp)^41,42^. Furthermore, the large jumps of p53 along DNA may enable the skipping of the molecules bound to DNA and searching of the target located nearby, and the jumps may reduce the target search time to ~ 90% (Supplementary text). Accordingly, the rotation-uncoupled movement and jumps of p53 may contribute to the target search by extending the search distance.

## Materials and methods

### Fluorescence microscopy

A fluorescence microscope was constructed as described in our earlier report^20^, with some modifications. The output of the 532-nm laser (CL532-050-L; CrystaLaser, Reno, NV, USA) was magnified tenfold and was focused on the back focal plane of an oil-immersion objective lens (N.A. = 1.40; Nikon, Tokyo, Japan) using a spherical lens (*f* = 500 mm). A dichroic mirror (Di01-R405/488/532/635–25 × 36; Semrock, Inc., Rochester, NY, USA), located between the two lenses, was used to introduce the light to the objective lens. The flow cell was placed above the objective lens. In the back focal plane of the objective lens, the radial distances between the lens center and the excitation laser focus were 2.63, 2.68, and 2.73 mm for HILO, critical angle TIRF, and TIRF, respectively. The corresponding incident angle for HILO was 59.6°, which was smaller than the critical angle of the total internal reflection (60.7°). The incident angle for critical angle TIRF was 61.4°, which was close to the critical angle. The incident angle for TIRF was 63.5°, which was larger than the critical angle. The fluorescence from the sample was collected using the same objective lens, passed through the dichroic mirror, and then passed through the spatial filter unit composed of two multiple lenses (*f* = 200 and 70 mm) and a slit (Optonica Co., Kyoto, Japan). The slit width was set between 0.8 and 1.5 µm, such that a single DNA could be selected in the observation area (600 × 10 ~ 19 pixels). The width of the point spread function of the fixed fluorescent spot was ~ 200 nm. In the flow channel, DNA fluctuated ~ 64 nm along the perpendicular axis against the flow. A 532-nm notch filter, a 532-nm long pass filter, and a 590-nm short pass filter were placed in the fluorescence detection path. A spherical lens (*f* = 70 mm) was used to focus the fluorescence on the TDI-EM CCD camera (Hamamatsu Photonics, KK, Hamamatsu, Japan). The final magnification of the image was 100×.

### Sample preparation

We used a thermostable and cysteine-modified mutant of human p53 (C124A, C135V, C141V, W146Y, C182S, V203A, R209P, C229Y, H233Y, Y234F, N235K, Y236F, T253V, N268D, C275A, C277A, and K292C)^20^. The expression and purification of p53 were conducted by following our reported method^20^. Briefly, p53 with the GST tag was expressed in *Escherichia coli* BL21 (DE3) pLysS cells. The cells were lysed by sonication, and the supernatants were loaded onto a GST column (GSTrap FF; GE Healthcare, Tokyo, Japan). The GST tag was cleaved using PreScission Protease (GE Healthcare), and samples were collected. The samples were purified further using a heparin column (HiTrap Heparin HP; GE Healthcare). DNA-binding ability of the purified sample was confirmed using a titration measurement based on fluorescence anisotropy^8^. The purified p53 was labeled with ATTO532 using maleimide chemistry, as reported in our previous study^20^. The p53 sample labeled with ATTO532 was purified using a cation exchange column (HiTrap SP HP; GE Healthcare). The labeling ratio was determined to be 0.8 dyes/monomer.

### Tethering of DNA to flow cell

To tether DNAs in the flow cell, we used DNA garden methods to produce arrays of stretchable DNAs^32^. Briefly, NeutrAvidin (Thermo Fisher Scientific, Waltham, MA, USA) was microcontact-printed on coverslips coated using an MPC polymer (Lipidure-CM; NOF, Tokyo, Japan) with a custom-made PDMS stamp (Fluidware Technologies, Saitama, Japan). Then, a flow cell was constructed by assembling double-sided tape, the coverslip, and the slide glass. The respective width and height of the flow path were ~ 3 and 0.1 mm, respectively. One of two holes of the slide glass was connected using a syringe through a tube^20^. For further coating of the flow cell, we used polyvinylpyrrolidone K15 (Tokyo Chemical Industry, Tokyo, Japan) and bovine serum albumin (BSA; Sigma-Aldrich, Tokyo, Japan). Finally, λDNA (New England Biolabs, Ipswich, MA, USA), annealed with 5′-GGGCGGCGACCT-biotin-3′ (Sigma-Aldrich), was immobilized on the NeutrAvidin printed area of the inner surface of the flow cell.

### Fast kymograph measurements of p53

We introduced the labeled p53 at 0.25–3 nM in a buffer containing 20 mM HEPES, 0.5 mM ethylenediaminetetraacetic acid (EDTA), 1 mM dithiothreitol, 0.5 mg/mL BSA, 2 mM Trolox, 2 mM MgCl_2_, and 25–150 mM KCl (pH 7.9) into the flow cell tethering DNAs using a syringe pump (Chemyx, Stafford, TX, USA). We first observed the larger area of the DNA array using two-dimensional imaging at an exposure time of 33 ms and a laser excitation power of 0.5 mW. Then, the slit was closed so as to detect only a single DNA, and fast kymograph measurements were conducted in TDI mode at an excitation power of 50 mW at 22 °C. Measurements were performed immediately after the dilution of labeled p53 from the stock solution at several tens of µM and completed within 50 min to prevent the dissociation of the tetramer into the dimers or monomers^43^. We obtained at least 600 kymographs consisting of 1,000 consecutive measurements of 0.5-ms exposures in each of the individual experimental conditions.

### Tracking of p53 molecules in the kymographs

Single-molecule trajectories were obtained by tracking the center of the fluorescence intensity distributions at each time step for the kymographs. First, we picked peaks whose maximum fluorescence intensity was larger than 5 standard deviations of the background intensity at all time steps. Second, the selected peak detected at a time step was connected to the peak detected in the next time step if the two peaks were located within 2 pixels. If more than two peaks were detected at the next time step, the closest peak was connected. If there were no candidate peaks detected in the next time step, the procedure was repeated in the two subsequent time steps. Less than four missing time steps were allowed to connect traces considering the photoblinking of the fluorescence dye. Third, trajectories with at least five data points were selected. Finally, we determined the position of the molecule at every time step for each trajectory by fitting of the fluorescence distributions with a Gaussian function.

### MSD analysis

We calculated the MSDs of trajectories whose lengths were greater than 7 ms.

### Jump analysis

We identified jump events for p53 by searching for the end point of a trajectory and the start point of a nearby trajectory based on the following criteria. We selected events for which the distance between the two points was larger than approximately the eightfold distance expected for sliding molecules having a diffusion coefficient of 0.13 µm^2^/s^20^ and was smaller than the distance expected for the free-flowing molecules by the bulk flow at 665.3 nm/ms. The flow rate was estimated assuming a laminar flow at 0.2 µm apart from the inner surface of the flow cell. The maximum allowed time interval between the two points was 15 ms.

### Residence time analysis

Before the residence time analysis of the single molecule traces, two or more traces separated by the identified jumps were connected. The residence time was calculated as the time difference between the start and end points of the trajectories. To reduce the effects of flowing molecules on the residence time distribution, we used all data, except for the initial data bins from 2 to 3 ms, in the fitting of the two exponentials (Supplementary Fig. S1). The histogram bin size was 1 ms in 50–150 mM KCl, whereas that in 25 mM KCl was 3 ms owing to the limited number of traces obtained.

## Supplementary information

Supplementary information.

## References

[1] Berg OG, Winter RB, Von Hippel PH (1981). Diffusion-driven mechanisms of protein translocation on nucleic acids. 1. Models and theory. Biochemistry.

[2] Von Hippel PH, Berg OG (1989). Facilitated target location in biological systems. J. Biol. Chem..

[3] Halford SE, Marko JF (2004). How do site-specific DNA-binding proteins find their targets?. Nucleic Acids Res..

[4] Schmidt HG, Sewitz S, Andrews SS, Lipkow K (2014). An integrated model of transcription factor diffusion shows the importance of intersegmental transfer and quaternary protein structure for target site finding. PLoS One.

[5] Hammar P (2012). The Lac repressor displays facilitated diffusion in living cells. Science.

[6] Normanno D (2015). Probing the target search of DNA-binding proteins in mammalian cells using TetR as model searcher. Nat. Commun..

[7] Joerger AC, Fersht AR (2007). Structure–function–rescue: The diverse nature of common p53 cancer mutants. Oncogene.

[8] Itoh Y (2016). Activation of p53 facilitates the target search in DNA by enhancing the target recognition probability. J. Mol. Biol..

[9] Wang YM, Austin RH, Cox EC (2006). Single molecule measurements of repressor protein 1D diffusion on DNA. Phys. Rev. Lett..

[10] Greene EC, Wind S, Fazio T, Gorman J, Visnapuu ML (2010). DNA curtains for high-throughput single-molecule optical imaging. Methods Enzymol..

[11] Tafvizi A, Mirny LA, van Oijen AM (2011). Dancing on DNA: Kinetic aspects of search processes on DNA. ChemPhysChem.

[12] Forget AL, Kowalczykowski SC (2012). Single-molecule imaging of DNA pairing by RecA reveals a three-dimensional homology search. Nature.

[13] Lee AJ, Wallace SS (2016). Visualizing the search for radiation-damaged DNA bases in real time. Radiat. Phys. Chem. Oxf. Engl..

[14] Cuculis L, Abil Z, Zhao H, Schroeder CM (2016). TALE proteins search DNA using a rotationally decoupled mechanism. Nat. Chem. Biol..

[15] Kamagata K, Murata A, Itoh Y, Takahashi S (2017). Characterization of facilitated diffusion of tumor suppressor p53 along DNA using single-molecule fluorescence imaging. J. Photochem. Photobiol. C Photochem. Rev..

[16] Ahmadi A (2018). Breaking the speed limit with multimode fast scanning of DNA by Endonuclease V. Nat. Commun..

[17] Tafvizi A (2008). Tumor suppressor p53 slides on DNA with low friction and high stability. Biophys. J..

[18] Tafvizi A, Huang F, Fersht AR, Mirny LA, van Oijen AM (2011). A single-molecule characterization of p53 search on DNA. Proc. Natl. Acad. Sci. USA.

[19] Leith JS (2012). Sequence-dependent sliding kinetics of p53. Proc. Natl. Acad. Sci. USA.

[20] Murata A (2015). One-dimensional sliding of p53 along DNA is accelerated in the presence of Ca(2+) or Mg(2+) at millimolar concentrations. J. Mol. Biol..

[21] Murata A (2017). One-dimensional search dynamics of tumor suppressor p53 regulated by a disordered C-terminal domain. Biophys. J..

[22] Subekti DRG (2017). The disordered linker in p53 participates in nonspecific binding to and one-dimensional sliding along DNA revealed by single-molecule fluorescence measurements. Biochemistry.

[23] Itoh Y, Murata A, Takahashi S, Kamagata K (2018). Intrinsically disordered domain of tumor suppressor p53 facilitates target search by ultrafast transfer between different DNA strands. Nucleic Acids Res..

[24] Kamagata K (2019). Rational design using sequence information only produces a peptide that binds to the intrinsically disordered region of p53. Sci. Rep..

[25] Kamagata K, Mano E, Ouchi K, Kanbayashi S, Johnson RC (2018). High free-energy barrier of 1D diffusion along DNA by architectural DNA-binding proteins. J. Mol. Biol..

[26] Khazanov N, Levy Y (2011). Sliding of p53 along DNA can be modulated by its oligomeric state and by cross-talks between its constituent domains. J. Mol. Biol..

[27] Terakawa T, Kenzaki H, Takada S (2012). p53 searches on DNA by rotation-uncoupled sliding at C-terminal tails and restricted hopping of core domains. J. Am. Chem. Soc..

[28] Terakawa T, Takada S (2015). p53 dynamics upon response element recognition explored by molecular simulations. Sci. Rep..

[29] Takada S (2015). Modeling structural dynamics of biomolecular complexes by coarse-grained molecular simulations. Acc. Chem. Res..

[30] Tempestini A (2018). Sliding of a single lac repressor protein along DNA is tuned by DNA sequence and molecular switching. Nucleic Acids Res..

[31] Bonnet I (2008). Sliding and jumping of single EcoRV restriction enzymes on non-cognate DNA. Nucleic Acids Res..

[32] Igarashi C (2017). DNA garden: A simple method for producing arrays of stretchable DNA for single-molecule fluorescence imaging of DNA binding proteins. Bull. Chem. Soc. Jpn..

[33] Blainey PC, van Oijent AM, Banerjee A, Verdine GL, Xie XS (2006). A base-excision DNA-repair protein finds intrahelical lesion bases by fast sliding in contact with DNA. Proc. Natl. Acad. Sci. USA.

[34] McKinney K, Mattia M, Gottifredi V, Prives C (2004). p53 linear diffusion along DNA requires its C terminus. Mol. Cell.

[35] Blainey PC (2009). Nonspecifically bound proteins spin while diffusing along DNA. Nat. Struct. Mol. Biol..

[36] Thompson RE, Larson DR, Webb WW (2002). Precise nanometer localization analysis for individual fluorescent probes. Biophys. J..

[37] Schreiber G (2002). Kinetic studies of protein–protein interactions. Curr. Opin. Struct. Biol..

[38] Ubbink M (2009). The courtship of proteins: Understanding the encounter complex. FEBS Lett..

[39] Sugase K, Dyson HJ, Wright PE (2007). Mechanism of coupled folding and binding of an intrinsically disordered protein. Nature.

[40] Spoerner M, Herrmann C, Vetter IR, Kalbitzer HR, Wittinghofer A (2001). Dynamic properties of the Ras switch I region and its importance for binding to effectors. Proc. Natl. Acad. Sci. USA.

[41] Ali Azam T, Iwata A, Nishimura A, Ueda S, Ishihama A (1999). Growth phase-dependent variation in protein composition of the *Escherichia coli* nucleoid. J. Bacteriol..

[42] Shivaswamy S (2008). Dynamic remodeling of individual nucleosomes across a eukaryotic genome in response to transcriptional perturbation. PLoS Biol..

[43] Rajagopalan S, Huang F, Fersht AR (2011). Single-molecule characterization of oligomerization kinetics and equilibria of the tumor suppressor p53. Nucleic Acids Res..

