# Supplementary material for: Transient binding and jumping dynamics of p53 along DNA revealed by sub-millisecond resolved single-molecule fluorescence tracking
Source: Sci Rep. 2020 Aug 13;10:13697. doi: 10.1038/s41598-020-70763-y (PMC7426816; doi:10.1038/s41598-020-70763-y)
Supplement: Supplementary file 1 — Supplementary information. [file 41598_2020_70763_MOESM1_ESM.pdf]

Supporting information

Transient binding and jumping dynamics of p53 along DNA revealed by sub-millisecond resolved single-molecule fluorescence tracking

Dwiky Rendra Graha Subekti[1,2ǂ], Agato Murata[1,2ǂ], Yuji Itoh[1], Satoshi Takahashi[1,2] and Kiyoto Kamagata[1,2,]*

[1]Institute for Multidisciplinary Research for Advanced Materials, Tohoku University, Katahira 2-1-1, Aoba-ku, Sendai 980-8577, Japan

[2]Department of Chemistry, Graduate School of Science, Tohoku University, Sendai 980-8578, Japan

ǂ equally contributed

*Corresponding author

Kiyoto Kamagata

Institute of Multidisciplinary Research for Advanced Materials, Tohoku University, Katahira 2-1-1, Aoba-ku, Sendai 980-8577, Japan

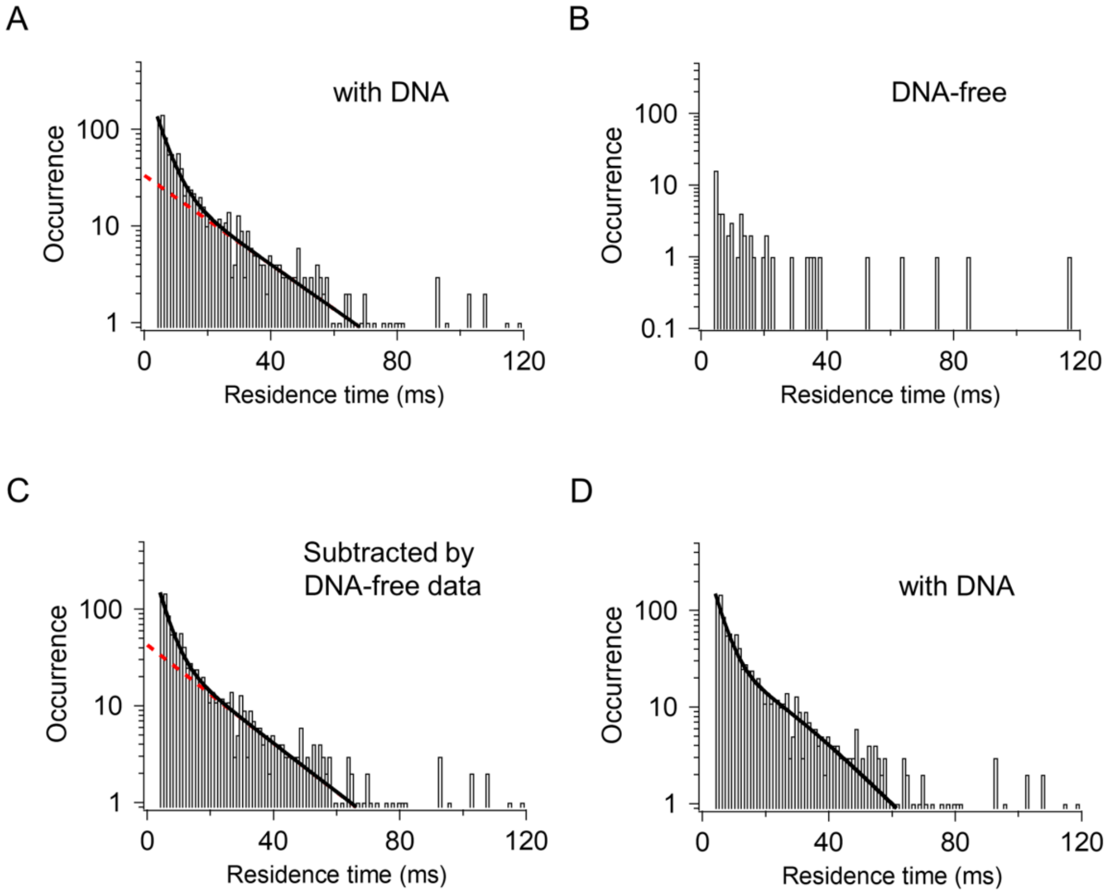

Fig. S1. Residence time distributions of p53 on DNA at 150 mM KCl obtained by using the critical-angle TIRF setup. The residence time distributions obtained in the presence (A, D), and absence (B) of DNA were compared. The panel (C) represents the distribution after the subtraction of the DNA-free data. A solid curve in panel (A) represents the best-fitted curve for the data excluding the initial time bin from 2 ms to 3 ms using the sum of two exponentials. A red dashed line represents the slow component of the fitted exponentials. The time constant and amplitude obtained by the fitting were 2.8±0.5 ms (91±2%) and 13±3 ms (9±2%), respectively, in which the errors were the

standard errors of three independent measurements. A solid curve in panel (C) represents the best-fitted curve using the sum of two exponentials. A red dashed line represents the slower component of the fitted exponentials. The time constant and amplitude obtained by the fitting were 2.3±0.5 ms (91±2%) and 11±3 ms (9±2%), respectively, in which errors were the standard errors of the three independent measurements. Thus, the parameters obtained by the fitting in panel (A) and those in panel (C) were similar each other within the errors. A solid curve in panel (D) represents the best-fitted curve for the data excluding the initial time bin from 2 ms to 3 ms using the sum of two exponentials corrected by the photobleaching effect (Eq. S3 and Supplementary text).

A

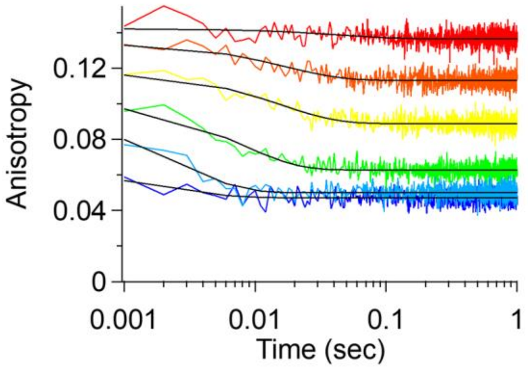

B

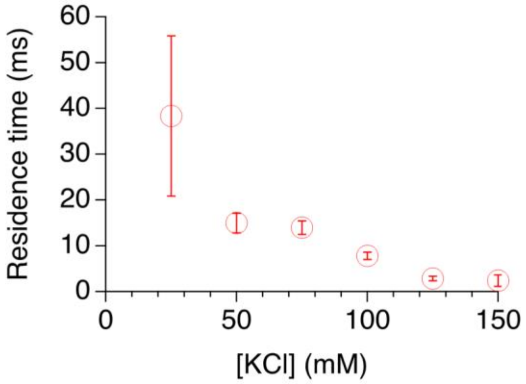

Fig. S2. Salt concentration dependence of the ensemble kinetic dissociation of p53 from DNA. (A) Time dependent changes of the fluorescence anisotropy for the dissociation reaction of the non-labeled p53 from the non-specific dsDNA labeled with FAM observed by using the stopped flow apparatus. The initial solution, containing one to one complex of p53 and DNA, was diluted by using the buffer containing various concentrations of KCl to initiate the dissociation of p53 from DNA upon the salt concentration jump (see Supplementary Text). The red, orange, yellow, green, cyan, and

blue traces represent the data obtained at the final KCl concentrations of 25, 50, 75, 100, 125, and 150 mM, respectively. Black curves are the best-fitted single exponentials. (B) KCl concentration dependence of the residence time of p53 obtained by the fitting.

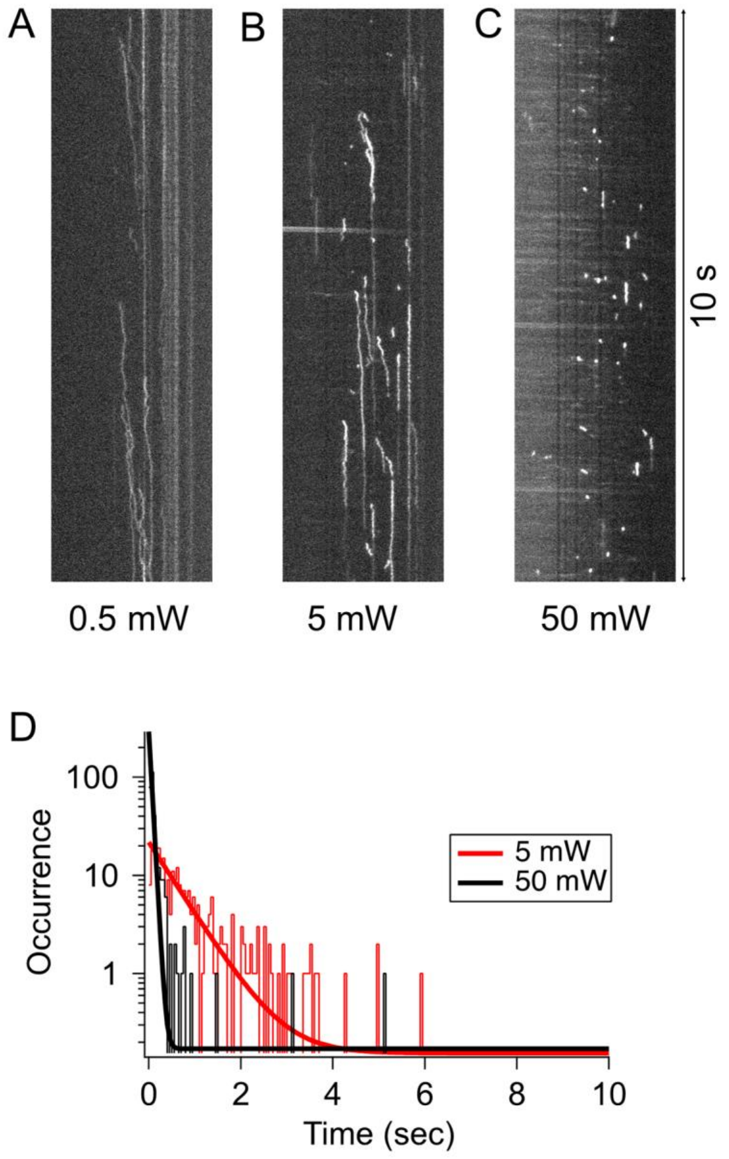

Fig. S3. Photobleaching of the fluorophore labeled to p53 observed at the high power excitation. Examples of the kymographs of p53 detected in the TIRF excitation geometry and by the TDI detection mode at the time resolution of 10 ms and at the excitation power of 0.5 mW (A), 5 mW (B), and 50 mW (C). White traces correspond to single p53 molecules. The experiments were conducted in the presence of 25 mM KCl

to elongate the residence time of p53 on DNA. (D) Distributions of the lengths of the tracked traces of p53 detected in the kymographs obtained at different excitation laser powers. The solid curves are the best-fitted single exponential functions giving the time constants of 591±28 ms and 52±1 ms for the 5 mW and 50 mW excitations, respectively.

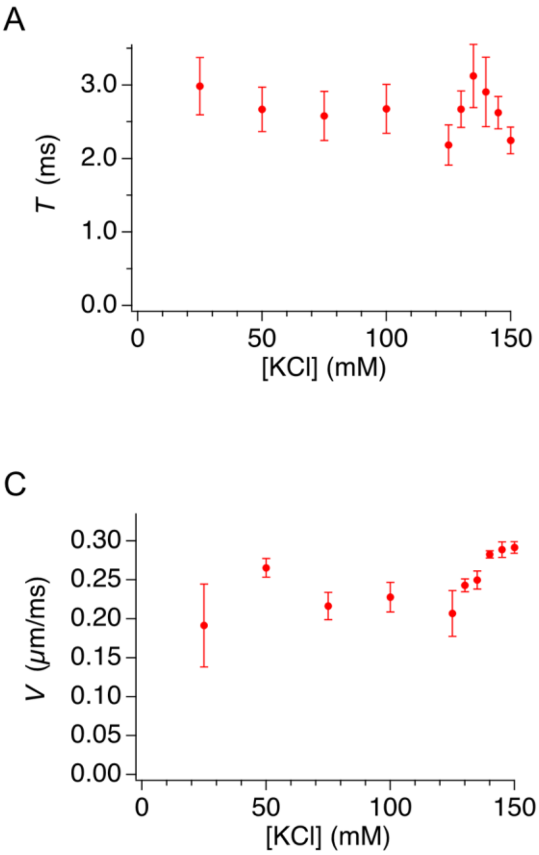

Fig. S4. Salt-concentration dependence of the various jump properties detected for p53 diffusing along DNA. The salt-concentration dependence of the averaged jump time (*T*) (A), the averaged jump length (*L*) (B), and the averaged jump velocity (*V*) (C) of p53. For each KCl concentration, at least 90 jump events were collected and used for the calculation of the properties presented. The error bars were the standard errors of 3 independent measurements. Except for the *L* value observed in 125 mM KCl, none of the observed values of *T*, *L*, and *V* was statistically different from the corresponding values observed in 50 mM KCl ($p > 0.05$, two-tailed *t*-test).

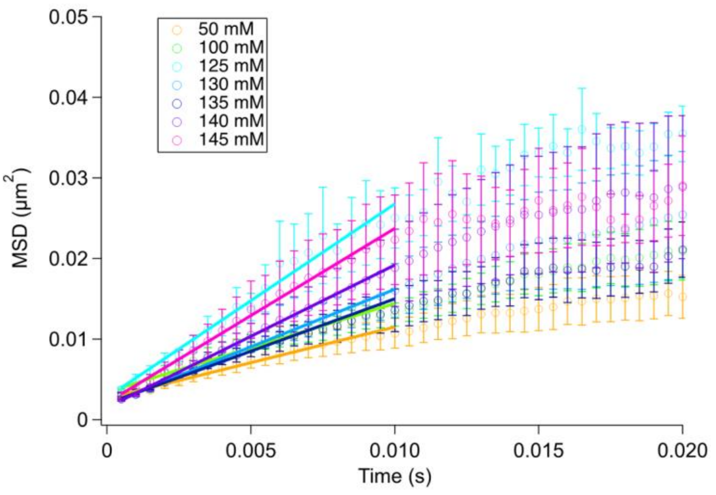

Fig. S5. Salt-concentration dependence of the 1D diffusion of p53 along DNA. The time courses of the mean square displacement (MSD) of p53 diffusing along DNA were presented that were observed in solutions containing the different concentrations of KCl. The error bars represent standard error of MSD points from average of at least 3 independent measurements. The straight lines showed the best fitted linear functions for the MSD data from 500 μs to 10 ms.

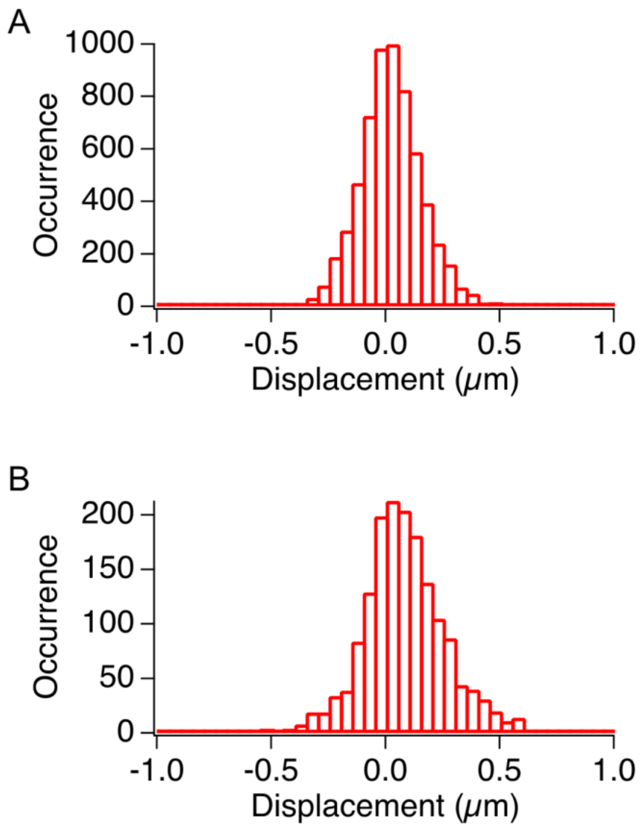

Fig. S6. Effect of the bulk flow on the movement of p53 along DNA. Displacement distribution of p53 diffusing along DNA in the time interval of 10 ms in the presence of 125 mM KCl (A) and 150 mM KCl (B). The drift of p53 along DNA by the flow was represented by the deviation of the displacement distribution center from 0. The drift in 150 mM KCl was larger than that in 125 mM KCl, but was still smaller than the distribution width corresponding to the diffusion.

Supplementary text

Calculation of the penetration depth and the illumination intensity in TIRF setup

The penetration depth of the evanescent wave in the TIRF setup affects the illumination of proteins bound to DNA which is located ~200 nm apart from the glass surface. In this section, we estimated the penetration depth, *d*, for the incident angles utilized in this study using the equation:

$$d = \frac{\lambda}{4\pi\sqrt{n_{\mathrm{glass}}{}^2 \times (\sin\theta)^2 - n_{\mathrm{solution}}{}^2}} \qquad \text{(S1),}$$

where λ, n_{glass}, n_{solution}, and θ are the wavelength of the incident light, the reflective index of the coverslip, the reflective index of the solution, and the incident angle, respectively. λ was 532 nm for the laser used in this study. n_{glass} was 1.5255 (± 0.0015) for the coverslip (Matsunami Glass Ind. Ltd., Osaka, Japan), and n_{solution} was 1.33. The incident angle was determined from the distance between the center of the incident laser and the center of the optical system. When θ was 63.5°, which was larger than the critical angle (60.7°), the penetration depth was calculated to be 137 nm. When θ was 61.4°, which was close to the critical angle, the penetration depth was calculated to be 268 nm.

We next estimated the illumination intensity at a perpendicular distance z from the glass surface, $I(z)$, using the equation:

$$I(z) = I(0)e^{-z/d} \qquad \text{(S2).}$$

where z, corresponding to the distance between DNA and the surface, was assumed to 200 nm [1]. When d was 137 nm corresponding to the TIRF condition with the incident angle of 63.5°, the illumination intensity was $0.233 \times I(0)$. In contrast, when d was 268 nm corresponding to the critical angle TIRF, the illumination intensity was $0.474 \times I(0)$. The critical angle TIRF can illuminate the molecules bound to DNA 2.0-fold more strongly than the TIRF with the incident angle of 63.5°, consistent with the signal intensity enhancement of the critical angle TIRF observed in Fig. 2 of the main text.

Measurement of the kinetic dissociation rates of p53 from DNA

To measure the residence time of p53 bound to non-specific DNA at the ensemble level, we conducted the stopped flow measurements of the fluorescence anisotropy changes by using 473 nm laser (Laser Quantum gem 473, Laser Quantum, Stockport, England) and stopped-flow mixing system (UNISOKU USP-203; UNISOKU,

Osaka, Japan) coupled with the polarized fluorescence detection[2]. We monitored the dissociation process of p53 from DNA as a decrease of fluorescence anisotropy of DNA labeled with a fluorescent dye after the mixing of the solution containing one to one complex of p53 and DNA and the buffer solution containing various concentrations of KCl (Fig. S2A). The initial solution of the one to one complex of p53 and dsDNA contained 40 nM p53 in the tetrameric form, 10 nM nonspecific dsDNA labeled with FAM, 20 mM HEPES, 25 mM KCl, 2 mM $MgCl_2$, 0.5 mM EDTA, 2 mM Trolox, and 0.1 mg/mL BSA at pH 7.9. The sequence for DNA was 5'-6-FAM-AATATGGTTTGAATAAAGAGTAAAGATTTG-3' (Sigma-Aldrich Co., Tokyo, Japan). The p53/DNA solution was diluted by the same buffer solution containing various concentrations of KCl at the volume ratio of 1:1 to initiate the dissociation reaction by the salt concentration jump. The fluorescence intensity time courses of FAM for each of the perpendicular ($I_{\perp}$) and parallel ($I_{\parallel}$) settings of the polarizers were monitored more than 3 times, averaged, and then converted into the anisotropy time courses. The mixing dead time of the stopped flow apparatus was ~1 ms. The measurements were conducted at 25°C. The fluorescence anisotropy curves were fitted with an exponential function, from which the residence times of p53 on DNA were obtained (Fig. S2B).

Correction of the photobleaching effect on the estimated residence time

To examine the effect of the bleaching on the estimated residence time, we built the model that considers the fast and slow dissociation kinetics of p53 from DNA and the disappearance of p53 on DNA by the photobleaching. We assumed that the p53 tetramer was labeled by four dyes and that the tracking of the sample on DNA was terminated by the bleaching of all the four dyes labeled. The residence time probability as a function of *t* was represented by the equation:

$$P(t) = (A_1 \exp\left(-\frac{t}{\tau_1}\right) + A_2 \exp\left(-\frac{t}{\tau_2}\right)) \times (1 - (1 - \exp\left(-\frac{t}{\tau_{\text{bleach}}}\right))^4) \qquad \text{(S3)},$$

where A_1, A_2, τ_1, τ_2, and τ_{bleach} denote the amplitudes for the two components, the residence times for the two components, and the time constant for the bleaching of the dye, respectively. The first and second halves of eq. S3 correspond to the probability of p53 molecules bound to DNA and the probability of p53 molecules having at least one dye without the photobleaching, respectively. We first fitted the residence time distribution data in 50 mM KCl (not shown) using eq. S3 and determined 18±1 ms as τ_{bleach}. The residence time of the slow component was not determined accurately,

because the photobleaching occurred faster than the slow dissociation and prevented the determination of the long residence time. In contrast, the residence time of the fast component was 2.0 ± 0.4 ms, consistent with 1.9 ± 0.4 ms obtained by the fitting using the two dissociation kinetics without photobleaching. We next fitted the data in 150 mM KCl using eq. S3 with the fixed τ_{bleach} value of 18 ms obtained in 50 mM KCl (Fig. S1D). The residence times of the fast and slow components were estimated as 2.2 ± 0.4 ms and 18 ± 7 ms, respectively. The obtained residence time of the slow component was not significantly different from 13 ± 3 ms that was obtained by the fitting using the two dissociation kinetics without photobleaching. Accordingly, the bleaching did not significantly affect the estimation of the residence times in 150 mM KCl. Furthermore, we obtained the similar results in the similar analysis conducted by assuming the labeling of 3 dyes per p53 tetramer, supporting smaller effect of the estimated residence times by the photobleaching.

Effect of jumps on the target search time

We first estimated the target search time, t_{search}, in the absence of the jump of p53 along DNA using the equation [3]:

$$t_{\mathrm{search}} = \frac{L_{\mathrm{DNA}} \times (t_{1\mathrm{D}} + t_{3\mathrm{D}}) \times 100}{L_{\mathrm{s}} \times TRP} \qquad \text{(S4)},$$

where L_{s}, $t_{1\mathrm{D}}$, $t_{3\mathrm{D}}$, L_{DNA}, and *TRP* are the search distance of p53, the residence time of the long-lived complex, average duration used for the 3D diffusion, the entire length of searchable DNA, and the target recognition probability, respectively. L_{DNA} was 3×10^{7} bp, assuming 1% of the accessible percentage of DNA. L_{s} was 700 bp obtained in this study. *TRP* of the activated p53 is 18% [4]. $t_{1\mathrm{D}}$ was 18 ms obtained in this study and $t_{3\mathrm{D}}$ was 60 ms [3]. Accordingly, the t_{search} value for one p53 molecule was estimated to be 310 min.

In the presence of the jump along DNA, we modified $t_{3\mathrm{D}}$ as the combination of the 3D diffusion and the jump. Considering the jump frequency, $t_{3\mathrm{D}}$ was corrected using the equation:

$$t_{3\mathrm{D\_corr}} = \frac{8 \times t_{3\mathrm{D}} + t_{jump}}{9} \qquad \text{(S5)},$$

where t_{jump} was the jump time (2 ms). $t_{3\mathrm{D\_corr}}$ was estimated 54 ms, slightly smaller than $t_{3\mathrm{D}}$. The t_{search} value for one p53 molecule was estimated 280 min by substituting the $t_{3\mathrm{D\_corr}}$ value for eq. S4. The results suggest that the jump of p53 along DNA may reduce

the target search time to ~90%.

References

1. Blainey, P. C., van Oijent, A. M., Banerjee, A., Verdine, G. L. & Xie, X. S., A base-excision DNA-repair protein finds intrahelical lesion bases by fast sliding in contact with DNA. *Proc. Natl. Acad. Sci. U. S. A.* **103**, 5752–5757 (2006).
2. Murata, A., *et al*., One-dimensional sliding of p53 along DNA is accelerated in the presence of Ca(2+) or Mg(2+) at millimolar concentrations. *J. Mol. Biol.* **427**, 2663–2678 (2015).
3. Kamagata, K., Murata, A., Itoh, Y. & Takahashi, S., Characterization of facilitated diffusion of tumor suppressor p53 along DNA using single-molecule fluorescence imaging. *J Photochem Photobiol C Photochem Reviews* **30**, 36–50 (2017).
4. Itoh, Y., *et al*. Activation of p53 facilitates the target search in DNA by enhancing the target recognition probability. *J. Mol. Biol.* **428**, 2916–2930 (2016).
